# Short-term outcomes of minimally invasive gastrectomy in population with obesity versus population without obesity: the obesity paradox

**DOI:** 10.1007/s13304-025-02144-x

**Published:** 2025-05-03

**Authors:** Marco Milone, Anna D’Amore, Gian Luca Baiocchi, Fabio Cianchi, Giovanni De Manzoni, Stefano De Pascale, Maurizio Degiuli, Giorgio Ercolani, Giovanni Ferrari, Laura Fortuna, Romario Uberto Fumagalli, Monica Gualtierotti, Federico Marchesi, Andrea Peri, Francesco Puccetti, Marco Realis Luc, Rossella Reddavid, Riccardo Rosati, Leonardo Solaini, Fabio Staderini, Marina Valente, Jacopo Viganò, Ugo Elmore, Simone Giacopuzzi

**Affiliations:** 1 Department of Clinical Medicine and Surgery, “Federico II” University of Naples, Via Sergio Pansini, 5, 80131 Naples, Italy; 2 Department of Clinical and Experimental Sciences, Surgery, University of Brescia, Brescia, Italy; 3 Digestive Surgery Unit, Careggi University Hospital, Florence, Italy; 4 General and Upper GI Surgery Division, Department of Surgery, University of Verona, Verona, Italy; 5 Digestive Surgery, European Institute of Oncology IRCCS, Milan, Italy; 6 Department of Oncology, University of Turin, San Luigi University Hospital, Orbassano, Italy; 7 Department of Medical and Surgical Sciences, University of Bologna, Bologna Italy / General and Oncologic Surgery, Morgagni - Pierantoni Hospital, AUSL Romagna, Forlì, Italy; 8 Minimally-Invasive and Oncological Surgical Department Niguarda Cancer Center, ASST Grande Ospedale Metropolitano Niguarda, Piazza Ospedale Maggiore, 3 20162, Milan, Italy; 9 Unit of General Surgery, University Hospital of Parma, Parma, Italy; 10 General Surgery II, Surgery Department, Fondazione IRCCS Policlinico S. Matteo, Pavia, Italy; 11 Department of Gastrointestinal Surgery, IRCCS San Raffaele Scientific Institute, 20132 Milan, Italy

**Keywords:** Gastric cancer, Gastrectomy, Minimally invasive surgery, Obesity, Paradox

## Abstract

This study aims to compare the short-term outcomes after minimally invasive gastrectomy between obese and non-obese population. Our analysis included data of 713 patients from ten departments of surgery. They were divided in non-obese group and obese group with 617 and 96 patients respectively. Significant differences were found in terms of mortality at 90 days (obese: 0 vs non-obese: 27, *p* = 0.037). Intraoperative data showed no significant differences in terms of conversion (obese: 4 vs non-obese: 43, *p* = 0.303). About postoperative complications, significant differences between the two groups were found only in terms of surgical infection (obese: 13 vs non-obese: 38, *p* = 0.009). About oncological outcomes, no differences were found about retrieved lymph nodes (obese: 30.71 ± 18.44 vs non-obese: 32.93 ± 17.62, *p* = 0.596) and about surgical radicality (R0) (obese:94 vs non-obese:594, *p* = 0.415). Obesity doesn’t worsen postoperative outcomes and minimally invasive gastrectomy in obese patients is related to a lower postoperative mortality.

## Introduction

Obesity is one of the major problems in Western countries and it could be considered a global pandemic [1–4]. Nevertheless, the current literature has showed an association between obesity and gastric cancer [5–10] and the increased prevalence of obesity has caused an increase even in the prevalence of gastric cancer.

Gastric cancer is the third leading cause of tumor-related deaths worldwide [11]. Its incidence and presentation depend on geographical area with the highest incidence seen in Eastern Asia, Eastern Europe and some Latin American countries [12–16]. In daily clinical practice, surgery remains the mainstay of therapy. In the 2013 annual Consensus Conference on gastric cancer, the Italian Research Group for Gastric Cancer (GIRCG) stated that minimal invasive surgery should be performed only for early gastric cancer (EGC) respecting the parameters of correct oncological radicality [17, 18]. However, the propensity of expert Italian upper gastrointestinal surgeons is performing minimally invasive techniques not only for early but also for advanced gastric cancer (AGC). Furthermore, the current literature also shows the safety of minimally invasive surgery for the treatment of gastric cancer [19].

Although minimally invasive approach presents well-known benefits, it could be technically more difficult in obese patients due to comorbidities related to obesity and few are known about feasibility and advantages of minimally invasive gastrectomy in obese patients [20–22]. Thus, is it safe for the treatment of gastric cancer in obese population? The aim of this study is to compare the short-term outcomes after minimally invasive gastrectomy between obese and non-obese population.

## Materials and methods

### Patients and protocol

Patients with gastric cancer who underwent a planned gastrectomy with D2 lymphadenectomy at 10 high-volume Italian centers for upper gastrointestinal surgery from January 1, 2017 to December 31, 2021 were retrospectively analyzed from prospectively maintained databases. Only patients who underwent a minimally invasive, both laparoscopic and robotic gastrectomy, were included in the study to compare the postoperative outcomes between the obese and non-obese population.

A written informed consent was obtained from each patient enrolled in the analysis. The study protocol conforms to the ethical guidelines of the 1975 Declaration of Helsinki.

The inclusion criteria were: age > 18 years, written informed consent provided, diagnosis of gastric cancer planned for elective gastric surgery. The exclusion criteria were age < 18 years, valid informed consent denied, locally advanced cancers not amenable to curative surgery or requiring en bloc multivisceral resection and severe systemic disease that contraindicated minimally invasive surgery.

All operations were performed by senior surgeons experienced in minimally invasive gastrectomy. To minimize the bias related to the presence of different surgeons, only procedures performed by experts of high-volume Italian centers for upper gastrointestinal surgery were considered.

Demographic information and surgery-related data were extracted. Demographic information included sex, age, BMI, American Society of Anesthesiologists ASA score, Charlson Comordibity Index, previous abdominal surgery, neoadjuvant therapy performed and tumor localization. Surgery-related data involved type of minimally invasive approach (laparoscopic or robotic) and type of surgical technique (subtotal or total).

### Outcomes

The enrolled patients were divided into two groups: obese (BMI ≥ 30 kg/m^2^) patient group and non-obese patient group.

The short-term outcomes of minimally invasive gastrectomy in obese versus non-obese patients were evaluated. These outcomes involved postoperative complications, mortality at 90 days, conversion, margin status and retrieved lymph nodes.

Furthermore, the complications were classified according to Clavien–Dindo (CD) Classification [23].

During postoperative course, patients were evaluated with clinical monitoring and daily blood tests. After the discharge, the patients were submitted to a check after 7, 30, 60 and 90 days.

### Statistical analysis

Statistical analysis was performed using the statistical package for the Social Sciences SPSS 28 system (SPSS Inc., Chicago, IL, USA). Continuous data were expressed as mean ± standard deviation. Categorical variables were expressed as percentages. Continuous variables were compared by the Mann–Whitney U test and categorical variables with the Chi-square χ2 test. All results are presented as two-tailed values and a *p* < 0.05 defined as a statistical significance.

## Results

Our analysis included data of 713 patients from ten departments of surgery. These patients were divided in non-obese group and obese group with 617 (86.5%) and 96 (13.5%) patients respectively. Patient and tumour characteristics are summarized in Table 1. No differences were found in terms of gender (*p* = 0.112), age (obese: 70.01 ± 11.2 vs non-obese: 70.07 ± 12.2, *p* = 0.624), Charlson Comordibity Index (*p* = 0.202), previous abdominal surgery (*p* = 0.27), neoadjuvant therapy (*p* = 0.527) and tumor localization (*p* = 0.930). Significative differences were found about ASA score (*p* = 0.024) with more ASA grades 3 and 4 cases in the obese group. About surgical approach, laparoscopic surgery was used in 609 (85.4%) cases (obese: 83 (13.6%) vs non-obese: 526 (86.4%)) and robotic surgery in 104 (14.6%) cases (obese:13 (12.5%) vs non-obese: 91 (87.5%)), (*p* = 0.877). About surgical technique, 490 (68.7%) patients (obese: 63 (12.9%) vs non-obese: 427 (87.1)) underwent subtotal gastrectomy and 223 (31.3%) patients (obese: 33 (14.8%) vs non-obese: 190 (85.2%)) underwent total gastrectomy (*p* = 0.48). Intraoperative data showed no significant differences in terms of conversion (obese: 4 (4.2%) vs non-obese: 43 (7%), *p* = 0.303). The analysis of postoperative complications showed significant differences between the two groups in terms of surgical infection (obese: 13 (13.5%) vs non-obese: 38 (6.2%), *p* = 0.009) while no differences interested the two group in terms of bleeding (obese: 2 (2.1%) vs non-obese: 41 (6.6%), *p* = 0.081), the need of ICU (obese: 13 (13.5%) vs non-obese: 71 (11.5%), *p* = 0.565), anastomotic leakage (obese: 7 (7.3%) vs non-obese: 31 (5%), *p* = 0.358), duodenal leakage (obese: 3 (3.1%) vs non-obese: 18 (2.9%), *p* = 0.911), anastomotic stenosis (obese: 1 (1%) vs non-obese: 7 (1.1%), *p* = 0.936), bowel obstruction (obese: 0 vs non-obese: 9 (1.5%), p = 0.234) and bowel perforation (obese: 2 (2.1%) vs non-obese: 9 (1.5%), *p* = 0.644), abdominal collection (obese: 6 (6.3%) vs non-obese: 33 (5.5%), p = 0.718), delayed gastric emptying (obese: 3 (3.1%) vs non-obese: 21 (3.4%), *p* = 0.888) and other major complications requiring re-intervention or other invasive procedures (obese: 2 (2.1%) vs non-obese: 21 (3.4%), *p* = 0.496). Considering the Clavien–Dindo Classification for postoperative complications, no significant differences were found in terms of CD-1 complications (obese: 9 (9.4%) vs non-obese: 69 (11.2%), *p* = 0.598), in terms of CD-2 complications (obese: 16 (16.7%) vs non-obese: 92 (14.9%), *p* = 0.655); in terms of CD-3 complications (obese: 10 (10.4%) vs non-obese: 61 (9.9%), *p* = 0.872); in terms of CD-4 complications (obese: 3 (3.1%) vs non-obese: 14 (2.3%), *p* = 0.609) and CD-5 complications (obese: 0 vs non-obese: 13 (2.1%), *p* = 0.151). Significant differences were found in terms of mortality at 90 days (obese: 0 vs non-obese: 27 (4.4%), *p* = 0.037); comparing this result with the CD-5 complications, it was found that in non-obese group other 14 deaths (2.3%) occurred within 90 days after discharge. About oncological outcomes, no differences were found about retrieved lymph nodes (obese: 30.71 ± 18.44 vs non-obese: 32.93 ± 17.62, *p* = 0.596) and about surgical radicality (R0) (obese:94 (97.9%) vs non-obese:594 (96.3%), *p* = 0.415). The short-term outcomes of minimally invasive gastrectomy in obese versus non-obese patients (postoperative complications, mortality at 90 days, conversion, margin status and retrieved lymph nodes) are summarized in Table 2.

**Table 1:**
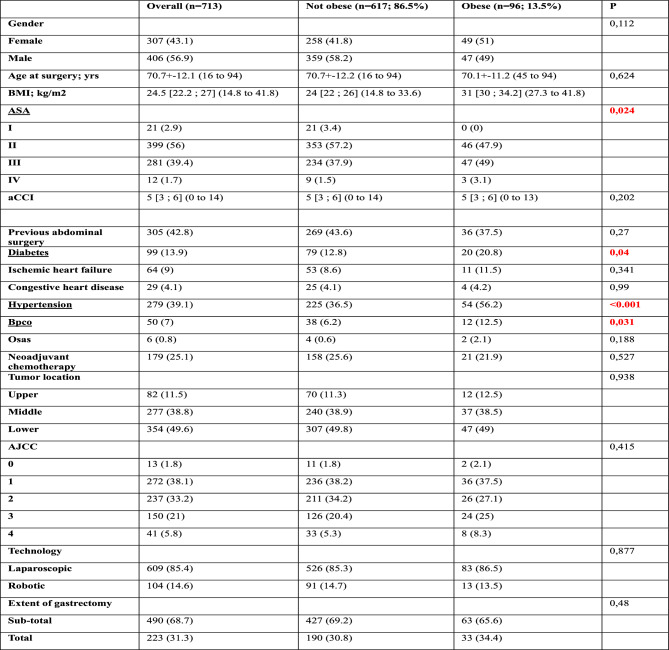
Demographic data and tumor characteristics

Values are expressed as number and (percentage)

Continuous variables are expressed as mean and standard deviation

*BMI* body mass index, *ASA* American Society of Anesthesiologists, *CCI* Charlson Comorbidity Index, AJCC 8th edition American Joint Committee on Cancer

**Table 2:** The short-term outcomes of minimally invasive gastrectomy in obese versus non-obese patients

	Overall (*n* = 713)	Not obese (*n* = 617; 86.5%)	Obese (*n* = 96; 13.5%)	*P*
Mortality at 90 days	27	27	0	0.037
Conversion	47	43	4	0.303
Margin status R0	688	594	94	0.415
Retrieved lymph nodes	32.63 ± 17.74	32.93 ± 17.62	30.71 ± 18.44	0.596
Clavien–Dindo classification				
0	424	367	57	0.970
1	78	69	9	0.598
2	108	92	16	0.655
3	71	61	10	0.872
4	17	14	3	0.609
5	13	13	0	0.151

Values are expressed as number and (percentage)

Continuous variables are expressed as mean and standard deviation

Short-term outcomes of minimally invasive gastrectomy in population with obesity versus population without obesity: the obesity paradox

In addition, patients were divided in subtotal minimally invasive gastrectomy group and in total minimally invasive gastrectomy group and each group has been divided in two subgroups (non-obese and obese group) to evaluate the aforementioned outcomes in obese versus non-obese patients. In detail, the subtotal minimally invasive gastrectomy group involved 490 patients (68.7%) (obese: 63 (12.9%) and non-obese: 427 (87.1%)) and the total minimally invasive gastrectomy group involved 223 patients (31.3%) (obese: 33 (14.8%) and non-obese: 190 (85.2%)). No differences were found in terms of short-term outcomes in obese versus non-obese patients in the subtotal minimally invasive gastrectomy group. On the contrary in the total minimally invasive gastrectomy group, significative differences were found in terms of CD-2 complications (obese: 8 (3.6%) vs non-obese: 20 (8.9%), *p* = 0.029).

## Discussion

Obesity could be considered a global pandemic and current literature showed an association between obesity and increased risk of gastric cancer [5–10]. In detail, excess body weight (BMI ≥ 25 according to the WHO classification for overweight and obesity) was associated with an increased risk of gastric cancer in non-Asian individuals [6], population where obesity was significantly associated with the risk of gastric cancer [7].

Current national trends in surgical treatment for gastric cancer showed that minimally invasive surgery was adopted for the treatment of both early and advanced gastric cancer and that the penetrance of minimally invasive approach correlated with volumes activity [19]. In 2013, we joined the annual Consensus Conference to gastric cancer, when Italian Research Group for Gastric Cancer (GIRCG) stated that minimal invasive surgery should be performed only for EGC respecting the parameters of correct oncological radicality [17, 18]. However, in the last decade, minimally invasive surgery has been increasingly used also for AGC with an improvement of recovery after surgery [24, 25] featuring less estimated blood loss, reduced use of analgesic injection, shorter hospital stay, decreased early and late complication. Recently, several randomized controlled trials (RCTs) [26–30] have revealed not only the surgical safety of laparoscopic gastrectomy for AGC but also that early morbidity rate was significantly lower after laparoscopic procedure in patients with AGC [29] showing a comparable long-term survival without an increase in recurrence and metastasis [28]. In this setting, we decided to perform a national survey on the current status of minimally invasive gastric practice [19] which has stated that surgeons are not used to properly respect the guidelines developed by GIRCG in 2013 and minimally invasive surgery was adopted for the treatment of both early and advanced gastric cancer. The survey [19] has also demonstrated that obesity didn’t impact on the choice to select a minimally invasive approach. This study aims to compare the short-term outcomes after minimally invasive gastrectomy between obese and non-obese population.

It is well known that the impact of obesity and the associated comorbidities, such as type 2 diabetes, hypertension, cardiovascular disease, obstructive sleep apnea syndrome, non-alcoholic fatty liver disease on the outcomes after major surgery, has increased a wide interest in surgery over the years. Additionally, obesity has always been considered as a disease that predisposes to technically more difficult interventions due to the excess adipose tissue and to the laparoscopic surgery limitations. Specifically, obesity with an excessive fatty tissue in abdominal wall and cavity could increase the difficulty of exposing the surgical field, further increasing the laparoscopic surgery limitations, such as two-dimensional images, decreased tactile sensation, magnification of physiological hand tremor, decreased dexterity due to instruments used and limited range of instrument motion [31, 32]. Previous studies have demonstrated that these difficulties, obesity-related, were associated with longer operative time, higher intra-operative blood loss, lower extent of lymph node dissection and a higher risk of postoperative complications [33–38], such as pancreatic fistula and anastomotic leakage [39]. However, our results showed no significant differences between the two groups in terms of intra-operative complications, conversions and oncological outcomes as shown by Tsekrekos A. et al. [40]. Of interest, significant differences were found about postoperative mortality at 90 days with 27 deaths in non-obese group and no death in obese group. About postoperative complications, significant differences were found only about surgical infection which appeared significantly higher in non-obese group; considering the Clavien–Dindo Classification for postoperative complications, no significant differences were found in terms of CD-1, CD-2, CD-3, CD-4 and CD-5 complications. These results might seem a paradox because our results not only suggested that obesity doesn’t worsen postoperative outcomes after minimally invasive gastrectomy performed by experienced surgeons but also that, in this setting, minimally invasive approach is related to a lower postoperative mortality. Of interest, these results also suggested safety and efficacy of minimally invasive surgery for the treatment of gastric cancer in obese patients because no significant differences were found in terms of postoperative complications, and about oncological outcomes, no differences were found about retrieved lymph nodes and surgical radicality (R0).

However, this study presents some limitations, such as its retrospective, non-randomized character and the different sample size of obese and non-obese group.

## Conclusions

Thus, no definitive conclusions can be drawn and it could be considered only a proof of concept and a call to perform ad hoc high-quality studies involving high-volume centers for upper gastrointestinal surgery to evaluate the postoperative outcomes after minimally invasive gastrectomy between the obese and non-obese population and how obesity could influence the postoperative outcomes.
